# Clinical evaluation of fully automated molecular diagnostic system “Simprova” for influenza virus, respiratory syncytial virus, and human metapneumovirus

**DOI:** 10.1038/s41598-020-70090-2

**Published:** 2020-08-11

**Authors:** Ikuyo Takayama, Shohei Semba, Kota Yokono, Shinji Saito, Mina Nakauchi, Hideyuki Kubo, Atsushi Kaida, Masashi Shiomi, Akihiro Terada, Kiyotaka Murakami, Katsushi Kaji, Keiichi Kiya, Yoshitaka Sawada, Kunihiro Oba, Sadasaburo Asai, Toshihiro Yonekawa, Hidetoshi Watanabe, Yuji Segawa, Tsugunori Notomi, Tsutomu Kageyama

**Affiliations:** 1 Influenza Virus Research Center, National Institute of Infectious Diseases, Tokyo, Japan; 2 Eiken Chemical Co., Ltd., Tokyo, Japan; 3 Division of Microbiology, Osaka Institute of Public Health, Osaka, Japan; 4 Aizenbashi Hospital, Osaka, Japan; 5 Nakano Children’s Hospital, Osaka, Japan; 6 Nishi-Tokyo Central General Hospital, Tokyo, Japan; 7 Department of Pediatrics, Showa General Hospital, Tokyo, Japan; 8 Asai Children’s Clinic, Osaka, Japan

**Keywords:** Microbiology, Health care

## Abstract

Influenza virus, respiratory syncytial virus, and human metapneumovirus commonly cause acute upper and lower respiratory tract infections, especially in children and the elderly. Although rapid antigen detection tests for detecting these infections have been introduced recently, these are less sensitive than nucleic acid amplification tests. More recently, highly sensitive point-of-care testings (POCTs) have been developed based on nucleic acid amplification tests, which are easy to use in clinical settings. In this study, loop-mediated isothermal amplification (LAMP)-based POCT “Simprova” to detect influenza A and B viruses, respiratory syncytial virus, and human metapneumovirus was developed. Simprova system is fully automated and does not require skilled personnel. In addition, positive results can be achieved faster than with PCR. In this study, the accuracy of the POCT was retrospectively analyzed using 241 frozen stocked specimens. Additionally, the usability of the Simprova at clinical sites was assessed in a prospective clinical study using 380 clinical specimens and compared to those of real-time PCR and rapid antigen detection test. The novel LAMP-based POCT demonstrated high sensitivity and specificity in characterizing clinical specimens from patients with influenza-like illnesses. The Simprova is a powerful tool for early diagnosis of respiratory viral infections in point-of-care settings.

## Introduction

Respiratory tract infections, especially lower respiratory tract infections, are associated with high morbidity and mortality. Over 65% of respiratory infections are caused by viruses^1^ and the World Health Organization (WHO) has estimated that 3.9 million people succumb to acute respiratory viral infections every year^2^. Influenza virus (IV), respiratory syncytial virus (RSV), and human metapneumovirus (hMPV) infections are the most common causes of acute upper and lower respiratory tract infections such as pneumonia and bronchiolitis, and lead to hospitalization, especially in children and the elderly^3–6^. Timely diagnosis of these infections is important in the clinical management of patients and for the reduction in healthcare costs^7^. Early diagnosis can also avoid nosocomial spread of these viruses and the unnecessary use of antibiotics^7–9^. However, the clinical signs and symptoms of these viruses are similar, and it can be difficult to distinguish the causative viruses^1,10^. In addition, not only viruses but also bacteria cause respiratory tract infections with almost indistinguishable clinical symptoms^11^. Early methods of virus diagnosis mainly utilized time-intensive viral cultures,however, over the past several decades, these methods have evolved to provide more rapid results with easy-to-use rapid antigen detection test (RADT) based on immunochromatographic assay. Nowadays, RADT is widely available for diagnosis of IV in clinical settings. RADTs for RSV are also available, and has been reported that their sensitivity is comparable to that of RADT for IV^12^. In contrast, the available number of RADTs for hMPV is low because hMPV was first identified in 2001^13^. RADTs can be used in point-of-care (POC) settings, but they are less sensitive than molecular diagnostic tests such as PCR and other nucleic acid amplification tests (NAATs)^14,15^. More recently, highly sensitive point-of-care testings (POCTs) based on NAAT that are easy to use in clinical settings have been developed. IV has been a primary driver of this evolution of diagnostic methods due to its annual global epidemics, the availability of antiviral therapy, which must be given early to have an effect^16^, and the constant threat of new pandemic strains. In 2015, the first molecular POCT based on NAAT for IV was cleared by the US Food and Drug Administration (FDA)^17^. Furthermore, in 2017, the FDA re-classified RADTs for IV from class I to II devices, and now requires RADTs for IV to meet specific minimum criteria for sensitivity and specificity. Consequently, especially in the United States, the number of RADTs has decreased and molecular POCTs based on NAAT have been introduced^17^. There is already a report that molecular POCT to detect IV is expected to reduce hospitalization and mortality rates, and it appears to be cost-effective^18^. The current molecular diagnostic test used in both laboratory and POC setting employs several methods, including isothermal nucleic acid amplification, real-time reverse transcription PCR (rRT-PCR), nested multiplex PCR, and RT-PCR followed by hybridization and colorimetric visualization.

Recently, we developed a loop-mediated isothermal amplification (LAMP)-based POCT “Simprova” for the detection of respiratory bacteria^19^. The results of the POCT for detecting respiratory bacteria are obtained within 35 min after specimen collection with high sensitivity and specificity. Unlike common NAATs that require nucleic acid extraction and purification followed by amplification reaction, Simprova processed the entire steps automatically and can be used in POC settings. In addition, Simprova can diagnose multiple targets at once because it has a testing chip with multiple reaction wells.

In this study, Simprova for respiratory viruses (Simprova-RV) that contains lyophilized primers for detecting influenza A virus (IAV), influenza B virus (IBV), RSV, and hMPV was developed. The purpose of this study was to evaluate the performance of Simprova-RV. The accuracy of Simprova-RV for clinical specimens was assessed in a retrospective validation using frozen stocked specimen. Additionally, the usability of Simprova-RV at clinical sites was assessed in a prospective clinical study. rPCR was used as the gold standard test for comparison in the retrospective validation and the prospective clinical study.

## Material and methods

### In vitro-transcribed RNA controls

In vitro-transcribed RNAs were used to determine the analytical sensitivity of Simprova-RV assay. RNA transcripts for IV were prepared from the full-length of matrix (M) gene of A/Narita/1/2009 (H1N1)pdm09 (GISAID accession no. EPI180038) and non-structural (NS) gene of B/Massachusetts/02/2012 (EPI439259), and those for RSV were prepared from the full-length of nucleoprotein (N) gene of RSV/OsakaC.JPN/16.2012 (Genbank accession no. LC415429) and RSV/OsakaC.JPN/38.2011 (LC415430) as previously studied^20^. RNA transcripts for hMPV were prepared from the full-length of the N gene of hMPVA/OsakaC.JPN/13.2012 (LC510256) and hMPVB/OsakaC.JPN/14.2012 (LC510257). In vitro-transcribed RNAs were synthesized as previously studied^20^. The procedure is described in detailed below. The random hexamer primer was used for reverse transcription using a SuperScript III Reverse Transcriptase Kit (Thermo Fisher Scientific, Waltham, MA, USA) according to the manufacturer’s instructions. The entire coding region of each gene was amplified by PCR using Phusion High-Fidelity DNA Polymerase (New England BioLabs, Ipswich, MA, USA) with paired primers, with the reverse primer containing the T7 promoter sequence. RNA was transcribed using the T7 RiboMAX Express Large Scale RNA Production System (Promega, Madison, WI, USA), and treated with TURBO DNase (Thermo Fisher Scientific) to degrade the template DNA. The dNTPs and NTPs were removed using MicroSpin G-25 Columns (GE Healthcare, Piscataway, NJ, USA) according to the manufacturer’s instructions. The transcribed RNAs were quantified using a NanoDrop spectrophotometer (Thermo Fisher Scientific), and the absorbance value was used to calculate the copy numbers of the transcribed RNAs. The integrity of each transcribed RNA was assessed with a 2100 BioAnalyzer (Agilent Technologies, Santa Clara, CA, USA).

### Clinical specimens

**Figure 1:**
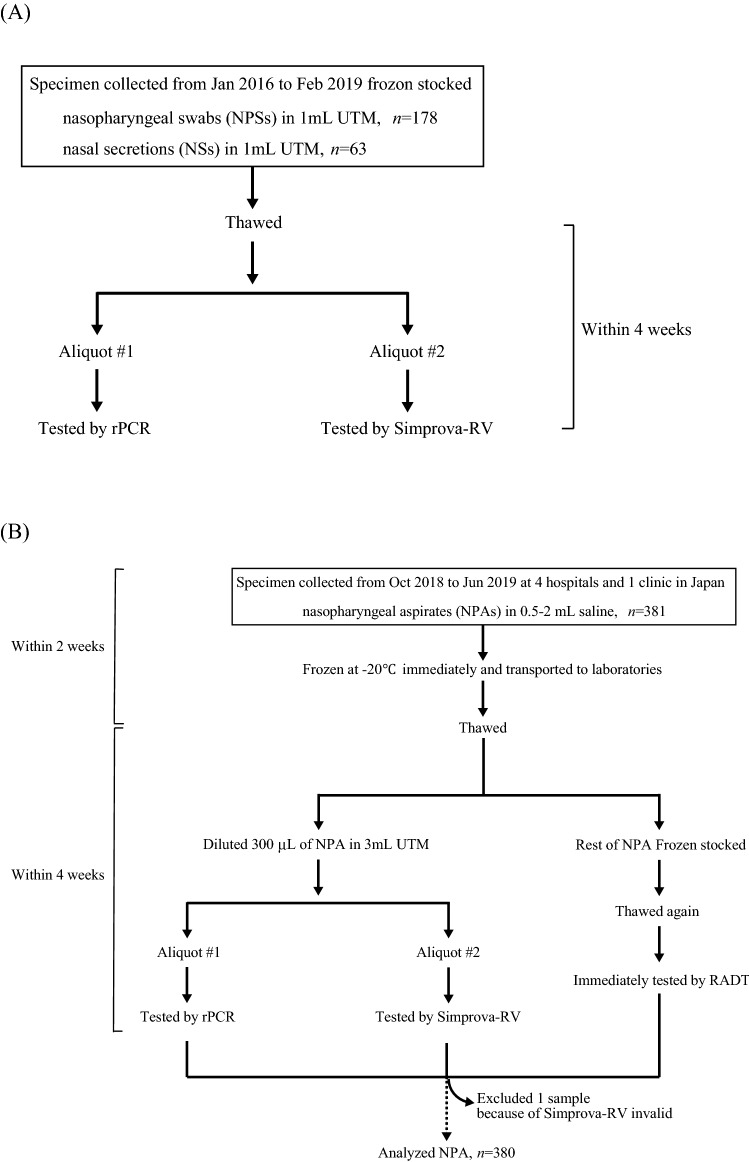
Overview of clinical specimen collection and diagnosis testing in a retrospective validation of Simprova-RV (**A**) and a prospective clinical study (**B**).

A flowchart of clinical specimen collection is given in Fig. 1. For a retrospective validation of Simprova-RV using frozen stocked specimen, 178 nasopharyngeal swabs (NPSs) and 63 nasal secretions (NSs) collected from patients who presented with influenza-like illnesses from January 2016 through February 2019 were used. All NPSs and NSs were collected in 1 mL of universal transport medium (UTM; Copan, Brescia, Italy) and stored at − 80 °C after initial analysis. rPCR and Simprova-RV were performed within 4 weeks of thawing a specimen.

For a prospective clinical study, 381 nasopharyngeal aspirates (NPAs) were collected from pediatric patients (age, ≦ 15 years) with influenza-like illnesses from October 2018 to June 2019 at 4 different hospitals and 1 clinic. NPA was collected with 0.5–2 mL of sterile normal saline because it is viscous, tenacious, and small, and difficult to handle. After sampling, NPA was frozen at − 20 °C immediately, and transported to the laboratories within 2 weeks. At the laboratories, 300 µL of NPA was diluted in 3 mL UTM (BD, Franklin Lakes, NJ, USA) and refrigerated; rPCR and Simprova-RV were performed within 4 weeks after thawing a specimen. The remaining NPA was stored at − 80 °C, and RADT was performed immediately after re-thawing of the NPA.

This study was conducted in accordance with the Declaration of Helsinki. This study was approved by the institutional medical ethical committees of Aizenbashi Hospital (#18-8), Nakano Children’s Hospital (#40), Nishi-Tokyo Central General Hospital (#2018-002), Showa General Hospital (#REC-094), the National Institute of Infectious Diseases (#946), Osaka Institute of Public Health (#1810-01), and Eiken Chemical Co., Ltd (#82-012). Participants or the parents of participants provided written informed consent.

### Testing by Simprova-RV

Simprova-RV was performed using a principal machine in which the pretreatment (extraction and purification of nucleic acid from the sample) part and the LAMP reaction and detection part were separated in this study. Simprova-RV was carried out as described previously^19^. In the pretreatment part, viral RNA was extracted and purified from 200 µL of specimen in UTM. Then, the viral RNA solution was manually injected into the testing chip and transferred to the LAMP reaction and detection part. Turnaround time for one test including extraction and purification of nucleic acid from the sample is approximately 30 min. Each target has multiple reaction wells including one well for positive control to check reactivity of the LAMP reagent on a testing chip. If an amplification signal was detected from at least one of the wells on the testing chip, the target was determined as “positive”.

### Testing by rPCR

Viral RNA for testing by rPCR assay was extracted by a MagMAX CORE Nucleic Acid Purification Kit (Thermo Fisher Scientific) using 200 µL of UTM mixed with clinical specimens according to the manufacturer's instructions, with an elution of 50 µL. cDNA was synthesized with a random hexamer primer using the PrimeScript RT reagent kit (Takara Bio, Shiga, Japan) and 10 µL viral RNA. Multiplex rPCR was conducted using the QuantiTect multiplex PCR kit (Qiagen, Hilden, Germany) for detecting IAV and IBV, RSV A and B, and hMPV as described previously^21^. The rPCR was performed in a 20 µL reaction containing 5 µL cDNA as the template and using the LightCycler 480 II (Roche, Basel, Switzerland). The RSV A and RSV B results were not separate, but were judged comprehensively and used as RSV results in this study.

### Rapid antigen detection test (RADT)

The Prime check Flu·RSV and Prime check hMPV (Alfresa Pharma, Osaka, Japan) that are commercially available were carried out according to the manufacturer’s instruction. Briefly, NPA sample collected using a swab provided in the RADT kit was added to an extraction reagent. Each device was read through visual inspection by three people after 10 min of incubation at room temperature.

### Analytical sensitivity of Simprova-RV assay

The analytical sensitivity of Simprova-RV assay was assessed by testing serial dilutions of quantified in vitro-transcribed RNA in 6 or 12 replicates at each concentration. Nuclease-free water was used as the negative control. The diluted RNA or nuclease-free water was manually injected into the testing chip and transferred to the LAMP reaction part. To determine the limit of detection (LOD) at 95% probability, a probit regression analysis was performed using StatPlus software (version 2009, AnalystSoft, Walnut, CA, USA).

### Statistical analyses

The sensitivity, specificity, positive predictive value (PPV) and negative predictive value (NPV) of Simprova-RV assay, and RADT were calculated with the MedCalc free statistical calculator (https://www.medcalc.org, MedCalc Software bvba, Ostend, Belgium). The statistical significance of quantification cycle (Cq) values of rPCR between rPCR positive and Simprova-RV positive samples was calculated using Mann–Whitney nonparametric test using GraphPad Prism software (version 7.0, Graph Pad Software, La Jolla, CA, USA) as the data were found not to be normally distributed via the Shapiro–Wilk’s test using RStudio (version 1.1, RStudio, Boston, MA, USA). Comparison between the numbers of Simprova-RV and RADT positive results was assessed using the Chi-square test with GraphPad Prism software. A *P* value of less than 0.05 indicated significance.

## Results

### The LOD of Simprova-RV assay

The LOD of Simprova-RV assay was determined by testing serial dilutions of quantified in vitro-transcribed RNAs in 6 or 12 replicates. As shown in Table 1, the LOD of Simprova assay for the detection of IAV, IBV, and RSV was 1.08–2.99 RNA copies/µL. The LOD of Simprova assay for detection of hMPV was 10.5–18.4 RNA copies/µL, which was nearly 10 times lower than that of IV and RSV (Table 1). There were no false positive results observed for any of the negative control samples in either target, and no cross-reactivity between these targets (data not shown).

**Table 1:** Analytical sensitivity of Simprova-RV assay.

Target	In vitro-transcribed RNA	Number of positive testing chips/number of tests for each RNA								LOD (copies/µL)
		Concentration of RNA (copies/µL)								
		20	10	5	2.5	1.25	0.625	0.3125	0.15625	
IAV	A/Narita/1/2009 (H1N1)pdm09 M gene	12/12	12/12	12/12	12/12	12/12	9/12	4/6	2/6	1.08
IBV	B/Massachusetts/2/2012 NS gene	12/12	12/12	12/12	11/12	10/12	7/12	2/6	2/6	2.97
RSV	RSV/OsakaC.JPN/16.2012 N gene	12/12	12/12	12/12	11/12	9/12	3/12	2/6	0/6	2.99
	RSV/OsakaC.JPN/38.2011N gene	12/12	12/12	12/12	12/12	12/12	7/12	3/6	3/6	1.59
hMPV	hMPVA/OsakaC.JPN/13.2012N gene	12/12	11/12	9/12	6/12	3/12	1/12	1/6	1/6	18.4
	hMPVB/OsakaC.JPN/14.2012N gene	6/6	6/6	5/6	4/6	1/6	3/6	NT	NT	10.5

*NT* not tested.

### Retrospective validation of Simprova-RV

A flowchart of enrolled patients, clinical specimen collection, and diagnosis testing is given in Fig. 1. To assess the accuracy of Simprova-RV for clinical specimen, we conducted a retrospective validation of Simprova-RV using frozen stocked specimen, and calculated the sensitivity and specificity using rPCR as a gold standard. A total of 241 clinical specimens collected from 132 male and 109 female child patients who presented with influenza-like illnesses were used. The median patient age was 3 years (range 0–14 years) as shown in Table 2. The clinical specimens consisted of 178 NPSs and 63 NSs, with 37–54 rPCR positive specimens for each target virus. Table 3 summarizes the comparison of the results for Simprova-RV and rPCR. Simprova-RV had 100% specificity for all targets. The sensitivity of Simprova assay for the detection of IAV, IBV, and RSV was over 90%. For hMPV, the sensitivity of Simprova assay was 75.5%, which was slightly worse than that of IV and RSV (Table 3). Overall, based on the retrospective validation, there were no false-positive results for any of the negative specimens in either targets, and no cross-reactivity between these targets when using clinical specimens (data not shown).

**Table 2:** Demographic and specimen characteristics of 241 patients for a retrospective validation of Simprova-RV.

	*n* (%)
Total number	241
**Demographic feature**	
Gender male	132 (54.8)
Age, median years (range)	3 (0–14)
**Virus(es), according to rPCR**	
IAV	44 (18.3)
IBV	33 (13.7)
RSV	50 (20.7)
hMPV	45 (18.7)
IAV + IBV	2 (0.83)
IAV + RSV	1 (0.41)
IAV + hMPV	1 (0.41)
IBV + RSV	1 (0.41)
IBV + hMPV	1 (0.41)
RSV + hMPV	2 (0.83)
**Specimen**	
Nasopharyngeal swab (NPS)	178 (73.9)
Nasal secretion (NS)	63 (26.1)

**Table 3:** The accuracy of Simprova-RV for clinical specimen during a retrospective validation.

Target	No. of positive specimens tested by rPCR	No. of positive specimens tested by Simprova-RV	Simprova-RV	
			Sensitivity %^a^	Specificity %^a^
IAV	48	47	97.90%	100%
			(88.9–100%)	(98.1–100%)
IBV	37	35	94.60%	100%
			(81.8–99.3%)	(98.2–100%)
RSV	54	50	92.60%	100%
			(82.1–97.9%)	(98.1–100%)
hMPV	49	37	75.50%	100%
			(61.1–86.7%)	(98.1–100%)

*CI* confidence interval.

^a^95% CI.

### Prospective clinical study

**Figure 2:**
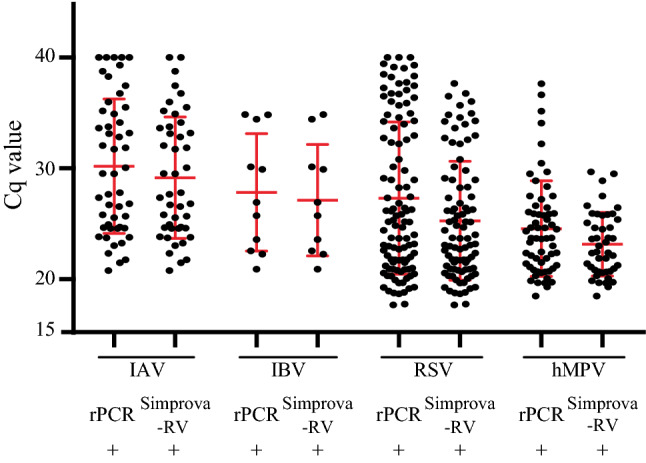
Comparison of rPCR Cq values between rPCR and Simprova-RV positive specimens in a prospective clinical study. Data present Cq value with the mean ± standard deviation (red line).

To assess the usability of Simprova-RV at clinical sites, we conducted a prospective clinical study. In this study, one invalid result was obtained with Simprova-RV by the negative results for the positive control, which was assumed to have arisen from a viscous and sticky specimen; the same sample was negative for all targets by rPCR (data not shown). This invalid specimen was excluded from the study analysis (Fig. 1). The demographic, clinical, and specimen characteristics of the analyzed patients in this study are shown in Table 4. During the study period, 380 NPAs were tested using Simprova-RV, rPCR, and RADT. The median patient age was 1 year (range 0–15 years) with 226 male and 154 female patients. Of the 380 patients, 275 (72.4%) were outpatients and 105 (27.6%) were hospitalized, 235 (61.8%) patients showed symptoms of lower respiratory tract infection, which was over 2 times the number of patients with upper respiratory tract infections, and 226 (61.1%) patients exhibited fever. The specimens of 190 (51.4%) patients were collected 72 h after symptom onset. Table 5 summarizes the results of Simprova-RV, rPCR, and RADT. Over the course of the study, rPCR detected 50 IAV positive, 11 IBV positive, 108 RSV positive, 61 hMPV positive, and 157 negative results. Two viruses were co-detected in 7 specimens; IBV and hMPV were in 2 specimens and RSV and hMPV were in 5 specimens (Table 4). Unlike rPCR, Simprova-RV detected 90.0% IAV and 90.9% IBV, but RADT detected 58.0% and 36.4%, respectively. For RSV, Simprova-RV detected 84.3% of RSV positive samples using rPCR, versus RADT detected 56.5% of them. For hMPV, Simprova-RV detected 73.8% of hMPV positive samples using rPCR, versus RADT detected 65.6% of them. Simprova-RV and RADT gave no false-positive result, and had 100% specificity and PPV for all targets. There are no associations between the clinical characteristics of patients and positive rate of viruses in each test (data not shown). The mean rPCR Cq values for rPCR and Simprova-RV positive specimens were 30.20 (range 20.79–40) and 29.16 (range 20.79–40), respectively for IAV; 27.83 (range 20.92–34.85) and 27.13 (range 20.92–34.85), respectively for IBV; 27.32 (range 17.68–40) and 25.27 (range 17.68–37.67), respectively for RSV; 24.57 (range 18.48–37.64) and 23.16 (range 18.48–29.68), respectively for hMPV (Fig. 2). Comparison of Cq values between rPCR and Simprova-RV positive specimens for all targets failed to disclose any significant difference (*P* = 0.06–0.80, Mann–Whitney nonparametric test). However, the rate of concordance results between rPCR and Simprova-RV were different by Cq values of the positive specimens (Fig. S1). Discordant results were observed when comparing specimens with Cq value of > 33 for IAV, IBV, and RSV, and those with Cq value of > 21 for hMPV. Since a slight difference in sensitivity was confirmed by detailed Cq value comparison, and it was found that genome of hMPV group B (hMPV B) collected during the prospective clinical study had mismatches in the LAMP primer region by sequencing analysis (data not shown), the hMPV LAMP detection primers were improved to detect hMPV B.

**Table 4:** Demographic, clinical and specimen characteristics of 380 analyzed patients in a prospective clinical study.

	*n* (%)
Total number	380
**Demographic feature**	
Gender male	226 (59.5)
Age, median years (range)	1 (0–15)
**Medical history**	
*Body temperature*	
Fever^a^	226/370 (61.1)
Data missing	10 (2.63)
*Time of visiting hospital or clinic from onset of illness*	
6 h >	24/370 (6.49)
6–12 h	26/370 (7.03)
12–24 h	51/370 (13.8)
24–72 h	79/370 (21.4)
72 h	190/370 (51.4)
Data missing	10 (2.63)
*Hospitalization*	
Outpatient	275 (72.4)
Inpatient	105 (27.6)
*Symptom*	
Upper respiratory tract infection	100 (26.3)
Lower respiratory tract infection	235 (61.8)
Other	45 (11.8)
**Virus(es), according to rPCR**	
IAV	50 (13.2)
IBV	9 (2.37)
RSV	103 (27.1)
hMPV	54 (14.2)
IBV + hMPV	2 (0.53)
RSV + hMPV	5 (1.32)

^a^Fever: defined as body temperature ≧ 38 °C

**Table 5:** Performance of Simprova-RV compared with the reference rPCR and RADT in a prospective clinical study.

Target	No. of positive specimens tested by rPCR		Simprova-RV^a^	RADT^a^
IAV	50	Sensitivity %	90.0% (78.2–96.7%)	58.0% (43.2–71.8%)
		Specificity %	100% (98.9–100%)	100% (98.9–100%)
		PPV %	100%	100%
		NPV %	98.5% (96.6–99.3%)	94.0% (91.9–95.6%)
IBV	11	Sensitivity %	90.9% (58.7–99.8%)	36.4% (10.9–69.2%)
		Specificity %	100% (99.0–100%)	100% (99.0–100%)
		PPV %	100%	100%
		NPV %	99.7% (98.3–100%)	98.1% (97.1–98.8%)
RSV	108	Sensitivity %	84.3% (76.0–90.6%)	56.5% (46.6–66.0%)
		Specificity %	100% (98.7–100%)	100% (98.7–100%)
		PPV %	100%	100%
		NPV %	94.1 (91.2–96.1%)	85.3% (82.4–87.8%)
hMPV	61	Sensitivity %	73.8% (60.9–84.2%)	65.6% (52.3–77.3%)
		Specificity %	100% (98.9–100%)	100% (98.9–100%)
		PPV %	100%	100%
		NPV %	95.2% (92.9–96.8%)	93.8% (91.5–95.6%)

*PPV* positive predictive value, *NPV* negative predictive value, *CI* confidence interval.

^a^95% CI.

### Improvement of hMPV LAMP detection primers

Simprova assay for hMPV using the new testing chip containing lyophilized improved detection primers was then performed. All positive specimens for hMPV and 5–10 each of positive specimens for other viruses by rPCR collected in the prospective clinical study were used for evaluation of improved hMPV LAMP detection primers. The numbers of positive specimens tested with Simprova-RV using the new testing chip with improved primers are shown in Table 6. The number of positive specimens for IAV, IBV, and RSV was almost same between old and new testing chips, but the number of them for hMPV increased from old (45) to new (58). The sensitivity of the improved Simprova assay for hMPV became 95.1% and LOD of that using in vitro-transcribed RNA became 1.90 copies/µL (data not shown).

**Table 6:** Number of positive specimens tested by Simprova for hMPV using improved testing chips.

Target	rPCR	Simprova-RV^a^	
		Old chip	New chip
IAV	5	5	5
IBV	7	6	6
RSV	10	10	9
hMPV	61	45	58
sensitivity %		73.8% (60.9–84.2%)	95.1% (86.3–99.0%)
specificity %		100% (86.3–100%)	100% (86.3–100%)

*CI* confidence interval.

^a^95% CI.

## Discussion

In this study, rapid, specific, and sensitive LAMP-based POCT for IAV, IBV, RSV and hMPV “Simprova-RV” was developed. Simprova-RV showed LOD of 1.08–18.4 RNA copies/µL (Table 1). These LODs were the same as the LOD of rRT-PCR as determined in previous studies^22,23^ or within 10 times lower.

To assess the accuracy of Simprova-RV for clinical specimen, we conducted a retrospective validation using frozen stocked specimens. Unlike that of rPCR, the sensitivity of Simprova assay for IAV, IBV, and RSV was over 90%, although that of Simprova assay for hMPV was 75.5% (Table 3). Overall, the retrospective validation indicated that there were no false-positive results and no cross-reactivity in either target. Simprova-RV has been shown to be able to provide accurate diagnosis of clinical specimens, indicated by the results of this retrospective validation.

Subsequently, we compared rPCR and RADT to assess the clinical usability of Simprova-RV. Nowadays, RADTs are widely used and popular at clinical sites. However, several systematic reviews have indicated poor sensitivity of RADTs; for example, the pooled sensitivity for IAV detection was 54.4% and that for IBV was 53.2%^24^. In this study, Simprova-RV showed high sensitivity for all targets, especially for IBV (Table 5), unlike RADTs. Differences between the number of positive results obtained with Simprova-RV and RADT were also compared at various times from the onset of illness (Table S1). Although previous reports have indicated that the sensitivity of RADT may be slightly lower in very early and later in the course of the disease^25^, in this study, the positive number shown by Simprova-RV was significantly higher than that by RADT in any duration of time (*P* = 0.00–0.02). Antiviral drugs for IV such as oseltamivir reduce symptoms and frequency of hospitalization if administered within 48 h of onset of symptoms, especially within 24 h^26^. Regardless of the time duration, highly sensitive Simprova-RV is a useful diagnostic tool that enables early treatment and prevention of infection spread. In the prospective clinical study, the sensitivity of Simprova assay for hMPV was slightly decreased than IV and RSV, but it was higher than that of RADT. Comparison of the rPCR Cq values between rPCR positive and Simprova-RV positive specimens for all targets showed that the Cq value ranges between the two groups were consistent for IAV and IBV, and there was no significant difference between the two groups for all targets (Fig. 2). In the prospective clinical study, the sensitivity of Simprova assay for all targets was slightly lower than that in the retrospective validation. For one reason, in the prospective clinical study, the virus concentration in the specimen was generally low because NPA was used instead of NPS. In fact, the rate of specimens with Cq ≥ 33 were higher in the prospective clinical study than in the retrospective validation for IV and RSV; 19/50 (38%) versus 12/48 (25%) for IAV, 3/11(27%) versus 6/37 (16%) for IBV, and 29/108 (27%) versus 13/54 (24%) for RSV, respectively (data not shown). Conversely, for hMPV, the rate of specimens with Cq ≥ 33 in the prospective clinical study was lower than that in the retrospective validation; 4/61 (7%) versus 5/49 (10%), respectively, even the sensitivity of Simprova-RV assay in the prospective clinical study was slightly lower than that in the retrospective validation. Additionally, the nucleotide sequence of hMPV B that was prevalence in the 2018/19 season was found to be different from that of hMPV LAMP detection primers. After the hMPV LAMP detection primers were improved to detect the prevalent hMPV B, the sensitivity of Simprova assay for hMPV became higher than the other targets. Thus, compared to RADT, NAAT is easier to improve against detection sensitivity degradation due to changes in genome sequence of prevalence viral strains.

In this Simprova-RV, multiple reaction wells are mounted on the testing chip, and multiple targets can be tested at the same time. In a previous study, we developed RT-LAMP assays to detect influenza A subtypes of H1pdm09 and H3 viruses and other respiratory viruses^20,27,28^. Therefore, by combining these assays, IVs can be easily and simultaneously identified with respect to type and subtype, and other respiratory viruses can also be detected.

In conclusion, the newly developed LAMP-based POCT “Simprova” for IV, RSV, and hMPV demonstrated high sensitivity and high specificity in clinical specimens from patients with influenza-like illnesses. This Simprova-RV processed the entire step automatically; the test can be performed without skilled personnel in the POC setting by simply placing a pretreatment cartridge, a testing chip, and the sample in the machine^19^. In addition, positive results can be achieved faster than by rPCR testing, because of the use of an isothermal nucleic acid amplification method. Furthermore, since multiple reaction wells are mounted on the testing chip, multiple targets can be tested at the same time. The Simprova-RV can be used to test for other pathogens and is a powerful tool for the POC setting.

## Supplementary information

Supplementary Information.
