# Publisher Correction: Clinical evaluation of fully automated molecular diagnostic system “Simprova” for influenza virus, respiratory syncytial virus, and human metapneumovirus

**DOI:** 10.1038/s41598-020-74039-3

**Published:** 2020-09-30

**Authors:** Ikuyo Takayama, Shohei Semba, Kota Yokono, Shinji Saito, Mina Nakauchi, Hideyuki Kubo, Atsushi Kaida, Masashi Shiomi, Akihiro Terada, Kiyotaka Murakami, Katsushi Kaji, Keiichi Kiya, Yoshitaka Sawada, Kunihiro Oba, Sadasaburo Asai, Toshihiro Yonekawa, Hidetoshi Watanabe, Yuji Segawa, Tsugunori Notomi, Tsutomu Kageyama

**Affiliations:** 1 Influenza Virus Research Center, National Institute of Infectious Diseases, Tokyo, Japan; 2 Eiken Chemical Co., Ltd, Tokyo, Japan; 3 Division of Microbiology, Osaka Institute of Public Health, Osaka, Japan; 4 Aizenbashi Hospital, Osaka, Japan; 5 Nakano Children’s Hospital, Osaka, Japan; 6 Nishi-Tokyo Central General Hospital, Tokyo, Japan; 7 Department of Pediatrics, Showa General Hospital, Tokyo, Japan; 8 Asai Children’s Clinic, Osaka, Japan

Correction to: *Scientific Reports* 10.1038/s41598-020-70090-2, published online 11 August 2020.

This Article contains a repeated error in the legends for Tables 3, 5 and 6 where,

“^*a*^95% CI”

should read:

“^*a*^( ): 95% CI”

