# Supplementary material for: Clinical evaluation of fully automated molecular diagnostic system “Simprova” for influenza virus, respiratory syncytial virus, and human metapneumovirus
Source: Sci Rep. 2020 Aug 11;10:13496. doi: 10.1038/s41598-020-70090-2 (PMC7419501; doi:10.1038/s41598-020-70090-2)
Supplement: Supplementary file 1 — Supplementary Information. [file 41598_2020_70090_MOESM1_ESM.docx]

Supplementary Materials for

Clinical Evaluation of Fully Automated Molecular Diagnostic System “Simprova” for Influenza Virus, Respiratory Syncytial Virus, and Human Metapneumovirus

Ikuyo Takayama^1*#^, Shohei Semba^2*#^, Kota Yokono^2^, Shinji Saito^1^, Mina Nakauchi^1^, Hideyuki Kubo^3^, Atsushi Kaida^3^, Masashi Shiomi^4^, Akihiro Terada^4^, Kiyotaka Murakami^5^, Katsushi Kaji^5^, Keiichi Kiya^6^, Yoshitaka Sawada^6^, Kunihiro Oba^7^, Sadasaburo Asai^8^, Toshihiro Yonekawa^2^, Hidetoshi Watanabe^2^, Yuji Segawa^2^, Tsugunori Notomi^2^ and Tsutomu Kageyama^1^

Fig. S1.

Fig. S1. Result concordance by Cq value between Simprova-RV and rPCR positive specimens.

Table S1. Number of positive specimens tested by Simprova-RV and RADT in a prospective clinical study, and the comparative analysis.
